# Protein restriction during pregnancy alters *Cdkn1c* silencing, dopamine circuitry and offspring behaviour without changing expression of key neuronal marker genes

**DOI:** 10.1038/s41598-024-59083-7

**Published:** 2024-04-12

**Authors:** Chiara Prodani, Elaine E. Irvine, Alessandro Sardini, Hannah J. Gleneadie, Andrew Dimond, Mathew Van de Pette, Rosalind John, Michelle Kokkinou, Oliver Howes, Dominic J. Withers, Mark A. Ungless, Matthias Merkenschlager, Amanda G. Fisher

**Affiliations:** 1 Epigenetic Memory Group, MRC LMS, Imperial College London, Hammersmith Hospital Campus, Du Cane Road, London, W12 0NN UK; 2 Metabolic Signalling Group, MRC LMS, Imperial College London, Hammersmith Hospital Campus, Du Cane Road, London, W12 0NN UK; 3 Whole Animal Physiology and Imaging, MRC LMS, Imperial College London, Hammersmith Hospital Campus, Du Cane Road, London, W12 0NN UK; 4 MRC Toxicology Unit, University of Cambridge, Tennis Court Rd, Cambridge, CB2 1QR UK; 5 Cardiff School of Biosciences, Cardiff University, Sir Martin Evans Building, Museum Avenue, Cardiff, CF10 3AX UK; 6 Psychiatric Imaging Group, MRC LMS, Imperial College London, Hammersmith Hospital Campus, Du Cane Road, London, W12 0NN UK; 7 MRC LMS, Imperial College London, Hammersmith Hospital Campus, Du Cane Road, London, W12 0NN UK; 8 Lymphocyte Development Group, MRC LMS, Imperial College London, Hammersmith Hospital Campus, Du Cane Road, London, W12 0NN UK; 9 Department of Biochemistry, University of Oxford, Oxford, OX1 3QU UK

**Keywords:** Behavioural methods, Bioluminescence imaging, Positron-emission tomography, Gene expression analysis, Mouse, Epigenetic memory, Imprinting, Intrauterine growth

## Abstract

We tracked the consequences of in utero protein restriction in mice throughout their development and life course using a luciferase-based allelic reporter of imprinted *Cdkn1c*. Exposure to gestational low-protein diet (LPD) results in the inappropriate expression of paternally inherited *Cdkn1c* in the brains of embryonic and juvenile mice. These animals were characterised by a developmental delay in motor skills, and by behavioural alterations indicative of reduced anxiety. Exposure to LPD in utero resulted in significantly more tyrosine hydroxylase positive (dopaminergic) neurons in the midbrain of adult offspring as compared to age-matched, control-diet equivalents. Positron emission tomography (PET) imaging revealed an increase in striatal dopamine synthesis capacity in LPD-exposed offspring, where elevated levels of dopamine correlated with an enhanced sensitivity to cocaine. These data highlight a profound sensitivity of the developing epigenome to gestational protein restriction. Our data also suggest that loss of *Cdkn1c* imprinting and p57^KIP2^ upregulation alters the cellular composition of the developing midbrain, compromises dopamine circuitry, and thereby provokes behavioural abnormalities in early postnatal life. Molecular analyses showed that despite this phenotype, exposure to LPD solely during pregnancy did not significantly change the expression of key neuronal- or dopamine-associated marker genes in adult offspring.

## Introduction

The transition from one cell-cycle-phase to another is regulated by activation and inhibition of heterodimeric cyclin/cyclin-dependent kinase (CDK) complexes^1,2^. These complexes are themselves regulated by cyclin-dependent kinase inhibitors (CKI), partners that can prevent or limit CDK activity^3,4^. Achieving a balance between quiescence and cell cycle progression is an essential feature of organismal biology. It is important for preserving stem cell populations as well as the differentiation of committed precursors, and deregulated in many types of cancer^5–8^.

The CiP/KiP family of CKIs, which includes p21, p27 and p57, are able to bind all CDK and cyclin subunits. They exert a major influence on cell cycle control and influence adult physiology, embryogenesis, and pathology, through diverse interactions that extend to factors that are not strictly cell cycle-associated^9,10^. Here we examine the impacts of dietary-induced p57^KIP2^ overexpression in utero, and in particular, the consequences this exposure has on the midbrain and behaviour of offspring. p57^KIP2^ is encoded by *Cdkn1c*, a maternally-expressed imprinted gene that lies within the imprinting cluster 2 (IC2) of mouse chromosome 7^11^. In development, *Cdkn1c* is expressed transiently, being particularly abundant in neural and skeleto-muscular tissue of mid-gestation embryos^12^ and marking cells that exit from proliferative cycles^13,14^. *Cdkn1c* is heavily implicated in regulating foetal growth and placental development^15–18^, is critical for tuning radial glial progenitor-mediated neuron output^8,19^ and can exert profound effects on physiology and behaviour when upregulated as little as two-fold^20–22^. Among the myriad of experimental phenotypes that are reported to arise from tissue-specific *Cdkn1c* deletion, or modest increases in *Cdkn1c* expression, many are assumed to reflect the role of p57^KIP2^ as a negative regulator of cell proliferation^13^. However, recently some studies have challenged this view, implicating *Cdkn1c* in promoting cortical development^23,24^ and showing that deletion of paternal alleles within the central nervous system can result in reduced neural stem and progenitor cell abundance, and a deficit in upper layer neurons of the cortex^25,26^.

Exposure to diets that are low in protein during pregnancy is known to enhance *Cdkn1c* expression in embryos, eroding DNA methylation across important regulatory regions, such as the somatic differentially methylated region (sDMR) of the locus^27–29^. Although the exact timing of this sensitivity to dietary challenge remains uncertain, monoallelic *Cdkn1c* expression is evident in mouse embryos from E6.5 onwards^30^, while pre-implantation exposure (E3.5) to low protein diet is reported to be sufficient to trigger a permanent reduction in neural stem cell numbers in the foetal brain^31^, as well as reducing bone growth at later gestational stages^32^. Early life exposure to low protein diet has also been linked to altered behaviour^29,33^, including changes in dopamine-dependent reward-processing and locomotor activity^27^. To longitudinally monitor allelic *Cdkn1c* expression in vivo, we previously developed a novel mouse reporter where imprinted gene expression can be visualised by bioluminescence imaging^28^. This model, where *Fluc* and *lacZ* genes are non-disruptively targeted into the 3’UTR of the endogenous *Cdkn1c* locus, uses T2A sites to simultaneously generate p57^KIP2^ (Cdkn1c), luciferase and β-galactosidase proteins from reporter-derived transcripts. With this reporter we previously showed that a low protein diet (LPD) fed to dams during pregnancy resulted in the loss of imprinting (LOI) of *Cdkn1c* in embryos, including in the midbrain, with sustained and inappropriate expression of paternal *Cdkn1c* into adulthood^28^.

To better understand the consequences of this exposure-induced loss of *Cdkn1c* imprinting, here we assess the behaviours and phenotypes of offspring as they mature postnatally from juveniles to adults. Our results show that exposure to LPD in utero generates offspring with increased numbers of tyrosine hydroxylase (TH)-positive neurons in the midbrain, altered dopamine circuitry and distinct behaviours. Despite this clear phenotype, LPD-exposed and non-exposed offspring showed broadly similar expression patterns of key marker genes in the brain. These results suggest that in utero exposure that elicits paternal *Cdkn1c* de-repression in the developing embryonic brain alters its longer-term function through a sustained increase in the abundance of TH-positive neurons.

## Results

### Paternal *Cdkn1c* is expressed selectively in embryos exposed to LPD in utero

**Figure 1:**
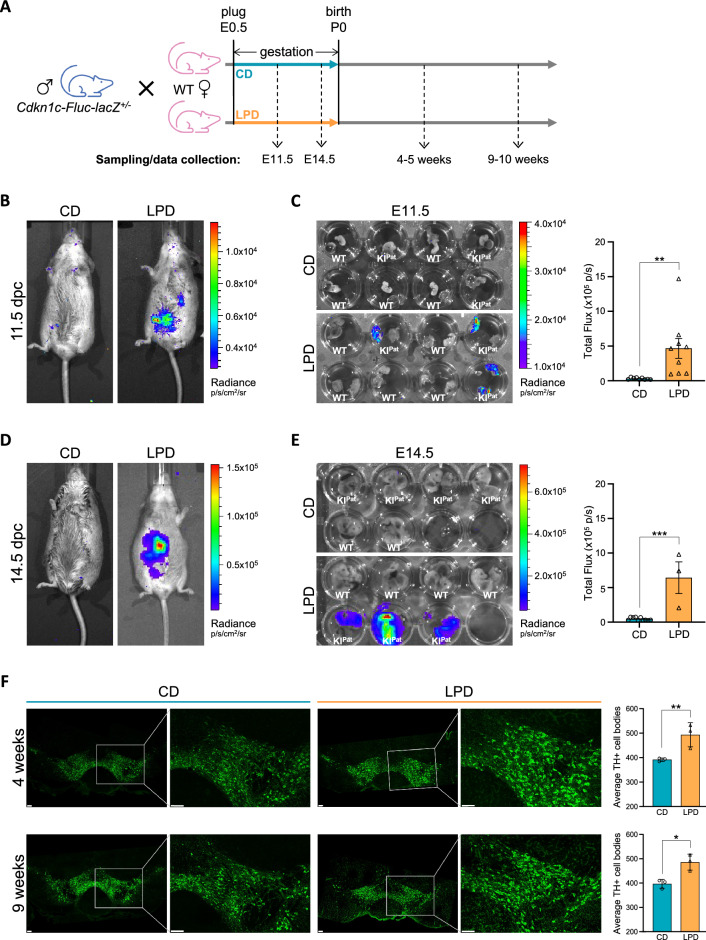
In utero exposure to LPD results in paternal *Cdkn1c* re-expression during embryonic development and elevated numbers of dopaminergic neurons in adult midbrain. (**A**) Schematic illustrating how *Cdkn1c-Fluc-lacZ* paternal knock-in (KI^pat^) and wildtype (WT) offspring were generated, together with the gestational dietary regimes used and the timepoints of experimental sampling. (**B**) Whole-body bioluminescent imaging of 11.5 dpc pregnant dams exposed to control diet (CD) or low protein diet (LPD) during pregnancy. (**C**) Ex-vivo bioluminescence imaging of KI^pat^ and WT E11.5 embryos and placentas exposed to CD or LPD during gestation. Graph (right) shows quantification of total flux in KI^pat^ embryos. CD n = 9, LPD n = 9; error bars = SEM; two-tailed unpaired t-test (**p = 0.0077). (**D**) Bioluminescence imaging of 14.5 dpc pregnant dams exposed to CD or LPD through pregnancy. (**E**) Ex-vivo bioluminescence imaging of KI^pat^ and WT E14.5 embryos and placentas exposed to CD or LPD during gestation. Graph (right) shows quantification of total flux in KI^pat^ embryos. CD n = 10, LPD n = 3; error bars = SEM; two-tailed unpaired *t*-test (***p = 0.0002). (**F**) Immunofluorescence detection of tyrosine hydroxylase (TH) positive cells (green) in representative midbrain tissue sections of juvenile and adult mice (4–5 and 9–10 weeks of age, respectively) exposed to CD or LPD in utero. Scale bars represent 100 µm. Right-hand graphs show the average number of TH positive cells in each condition. N = 3 animals per condition and ≥ 2 tissue sections averaged per animal; error bars = SD; Two-way ANOVA (diet p = 0.0007, age p = 0.9524, interaction p = 0.7399) with Sidak’s multiple comparisons test (**padj = 0.0079, *padj = 0.0159; two comparisons only).

We used the *Cdkn1c-Fluc-lacZ* reporter mouse line to track paternal *Cdkn1c* expression in vivo. This reporter line has been characterised in detail previously^28^, and was used here to visualise paternal *Cdkn1c* de-repression in embryos exposed to LPD. Briefly, wildtype (WT) females were mated with heterozygote (*Cdkn1c-Fluc-lacZ*^+/−^) male reporter mice, to generate offspring that were either WT, or inherited *Cdkn1c-Fluc-lacZ* paternally (KI^Pat^) (Fig. 1A). Pregnant dams were injected with the luciferase substrate D-luciferin, and whole-body imaging was used to detect and quantify bioluminescence signal (Fig. 1B). Consistent with *Cdkn1c* being almost exclusively maternally-expressed^11,25^, and correct paternal *Cdkn1c-Fluc-lacZ* silencing in developing embryos^28^, bioluminescence signal was not detected in pregnant females carrying WT or KI^Pat^ E11.5 embryos fed a normal control diet (CD). Bioluminescent signal was, however, detected in pregnant dams fed a calorie matched LPD (compare representative images shown in left and right panels of Fig. 1B). Among embryos isolated from these mice, bioluminescence was exclusively detected in KI^Pat^ foetuses derived from mothers fed LPD during pregnancy (Fig. 1C, left), and quantification of signal among all KI^Pat^ individuals confirmed a significant increase in LPD-exposed embryos as compared to controls (Fig. 1C, right). Similar results were seen at later stages of development (E14.5) in pregnant dams (Fig. 1D) and isolated embryos (Fig. 1E). These results are consistent with previous work^28^ showing that protein restriction during gestation induces paternally-derived *Cdkn1c* misexpression and LOI in embryos.

### Elevated numbers of midbrain TH-positive neurons in offspring exposed in utero to LPD

De-repression of paternal *Cdkn1c-Fluc-lacZ* initiated early in gestation can continue beyond birth and long after LPD exposure, whilst gestational sensitivity to LPD is ameliorated by supplementation of maternal LPD with folate, which effectively restores correct DNA methylation across the sDMR^28^. Independent studies by others have also shown that folate depletion in pregnancy impacts brain development in adults^27,31,34^ and that *Cdkn1c* upregulation can alter the proliferation and differentiation of developing dopaminergic neurons within the midbrain^20,27,29^. We therefore examined the brains and the behaviours of juvenile and adult offspring that had experienced gestational LPD exposure.

Tyrosine Hydroxylase (TH) is the rate-limiting enzyme in dopamine synthesis, and the most commonly used marker of dopaminergic neurons. As illustrated in Fig. 1F, a significant increase in the number of TH-positive neurons was detected in the midbrain of offspring at 4 and 9 weeks of age following in utero LPD exposure, compared to matched CD exposed offspring. Figure 1F (left) shows representative anti-TH immunofluorescence labelling (green) of midbrain sections at low magnification (with higher magnification insets) to enumerate TH-positive neurons. Quantification of the number of TH-positive cell bodies, provided in the accompanying plots (Fig. 1F, right), demonstrated that gestational exposure to LPD resulted in significant increases in the abundance of TH-neurons in the midbrain of reporter mice sampled at both 4 and 9 weeks of age (24% and 25% increases in TH-neurons, respectively).

### Altered behaviours in offspring previously exposed to LPD in utero

**Figure 2:**
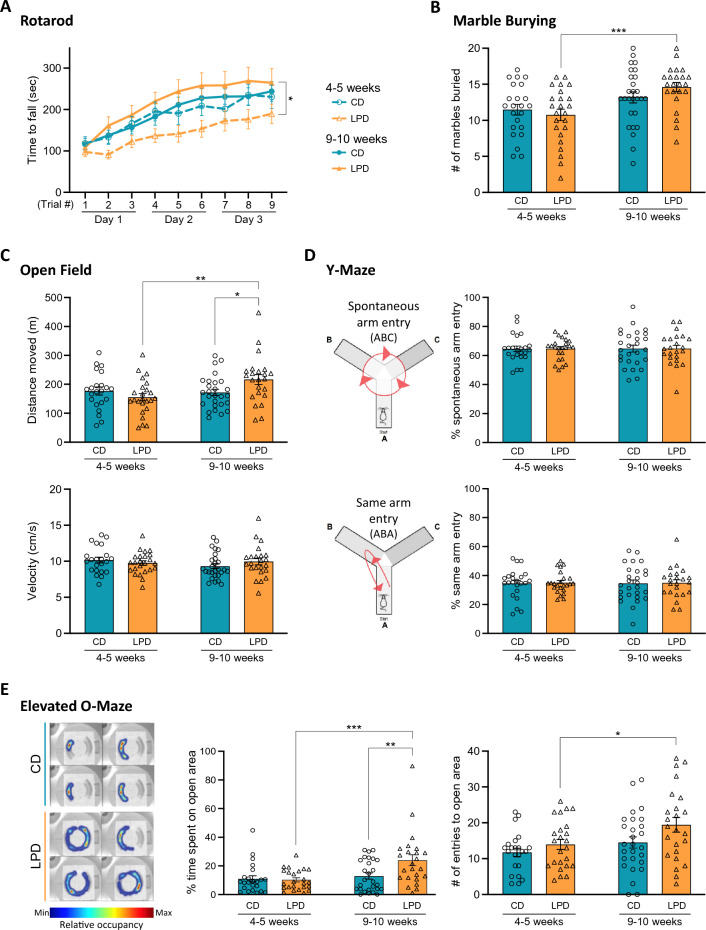
In utero exposure to LPD results in both transient and sustained changes in offspring behaviour. (**A**) Time course trials of rotarod latency (time to fall) in juvenile (4–5 weeks old, open symbols) and adult (9–10 weeks old, filled symbols) CD- (blue) or LPD- (orange) exposed mice. Two-way repeated measures ANOVA revealed a significant difference between the four groups (p = 0.0339). Sidak’s multiple comparisons test (main group effect) revealed a significant difference between LPD juveniles and adults (*padj = 0.0165; four pre-selected comparisons). CD 4–5 weeks n = 23, LPD 4–5 weeks n = 24, CD 9–10 weeks n = 27, LPD 9–10 weeks n = 23; error bars = SEM. (**B**) Analysis of marble burying in juvenile and adult CD- (blue) and LPD- (orange) exposed offspring, quantifying the number of marbles out of 20 which were > 2/3 buried after 20 min. Two-way ANOVA (age p = 0.0003, diet p = 0.6396, interaction p = 0.1494) with Sidak’s multiple comparisons test (***padj = 0.0009; two families of two comparisons). CD 4–5 weeks n = 23, LPD 4–5 weeks n = 24, CD 9–10 weeks n = 27, LPD 9–10 weeks n = 23; error bars = SEM. (**C**) Open field assessment of juvenile and adult CD- and LPD-exposed offspring over 60 min, quantifying total distance moved (upper) and velocity (lower). Two-way ANOVAs: distance (age p = 0.0405, diet p = 0.4028, interaction p = 0.0181), velocity (age p = 0.3943, diet p = 0.7514, interaction p = 0.1711); Sidak’s multiple comparisons tests (*padj = 0.0421, **padj = 0.0045; two families of two comparisons per analysis). CD 4–5 weeks n = 22, LPD 4–5 weeks n = 24, CD 9–10 weeks n = 27, LPD 9–10 weeks n = 23; error bars = SEM. (**D**) Y-maze performance of juvenile and adult CD- or LPD-exposed offspring, assessed as the percentage of spontaneous arm entries (ABC pattern, upper) and alternate arm entries (ABA pattern, lower). Two-way ANOVA analyses revealed no significant effects on %ABC entries (diet p = 0.9842; age p = 0.9404; interaction p = 0.9772) or %ABA entries (diet p = 0.8309, age p = 0.9866, interaction p = 0.9627). CD 4–5 weeks n = 23, LPD 4–5 weeks n = 24, CD 9–10 weeks n = 27, LPD 9–10 weeks n = 23; error bars = SEM. Schematics adapted from^75^ (CC BY 4.0; http://creativecommons.org/licenses/by/4.0/). (**E**) Elevated O-maze testing of juvenile and adult CD- or LPD-exposed mice. Representative occupancy heatmaps (left) illustrate movement of four individual CD and LPD adults. Open area occupancy was quantified by time spent (middle) or number of entries (right). Diet had a significant impact on open area occupancy by both measures (two-way ANOVAs: time spent (diet p = 0.0423, age p = 0.0028, interaction p = 0.0235), entries (diet p = 0.0271, age p = 0.0109, interaction p = 0.3957); Sidak’s multiple comparisons tests (*padj = 0.0347, **padj = 0.0044, ***padj = 0.0006; two families of two comparisons per analysis). CD 4–5 weeks n = 23, LPD 4–5 weeks n = 24, CD 9–10 weeks n = 27, LPD 9–10 weeks n = 24; error bars = SEM.

To gauge the behavioural impacts of in utero LPD exposure and paternal *Cdkn1c* mis-expression, we subjected juvenile (4–5 week old) and adult (9–10 week old) offspring to an array of tasks designed to evaluate basic motor functions, as well as anxiety-related and cognitive functions. Motor coordination, motor skill learning and balance were assessed using an accelerating rotarod, a tool often used to dissect motor capabilities from cognitive function^35,36^. LPD-exposed offspring underperformed in rotarod tests as compared to CD-exposed animals at 4–5 weeks of age (Fig. 2A, dashed lines) but interestingly, showed a significant improvement by 9–10 weeks of age (Fig. 2A, solid lines). This could be partially explained by LPD-exposed mice being slightly heavier than CD-exposed mice at 4 weeks of age, a difference which normalises as they mature (Supplementary Fig. S1A). While juvenile LPD and CD exposed animals showed similar marble burying capacity, a test that has often been used to assess dopaminergic and glutaminergic circuits^37^ or infer autistic-like behaviours^38^, a significant increase in this behaviour was evident in LPD-exposed offspring between 4–5 and 9–10 weeks of age, but not in CD-exposed controls (Fig. 2B). Open field testing showed that although movement was indistinguishable between juvenile LPD- or CD-exposed mice (4–5 weeks of age), increased locomotor activity was evident in the LPD group as they matured (Fig. 2C). In particular, an increase in distance moved (upper panel), rather than velocity (lower panel), was seen in LPD mice at 9–10 weeks. To investigate whether this increased activity might reflect enhanced exploratory behaviour, reduced anxiety, or short-term memory-related deficits, mice were subjected to Y-maze (Fig. 2D) and elevated O-maze (EOM) (Fig. 2E) testing. Y-maze assessment showed that the percentages of spontaneous alteration entries (ABC) (Fig. 2D, upper panel) and same arm entries (ABA) (Fig. 2D, lower panel) for animals over a 5 min test period were similar between juvenile and adult CD and LPD-exposed offspring. In contrast, in EOM tests, adult LPD-exposed offspring exhibited increased exploration (illustrated by representative heatmaps in Fig. 2E, left). In particular, LPD-exposed adults spent significantly more time in the open areas than age-matched CD-exposed mice or LPD-exposed juveniles (Fig. 2E, middle), and made significantly more open area entries as they matured (Fig. 2E, right). Dietary-exposure also had a significant overall effect on the distance travelled and velocity (Supplementary Fig. S1B), although individual pairwise comparisons were not significant for these metrics (see figure legends for details). Taken together these data show that offspring arising from in utero exposure to maternal LPD display specific behavioural phenotypes. Interestingly, underperformance in motor skill tests by juveniles, which could reflect a developmental delay in response to LPD, was fully restored as these mice matured. By 9–10 weeks, LPD-exposed offspring showed increased locomotor capacity and behaviours that might indicate a reduced level of anxiety relative to controls.

### Increased dopamine synthesis capacity and sensitivity to cocaine in LPD-exposed offspring

**Figure 3:**
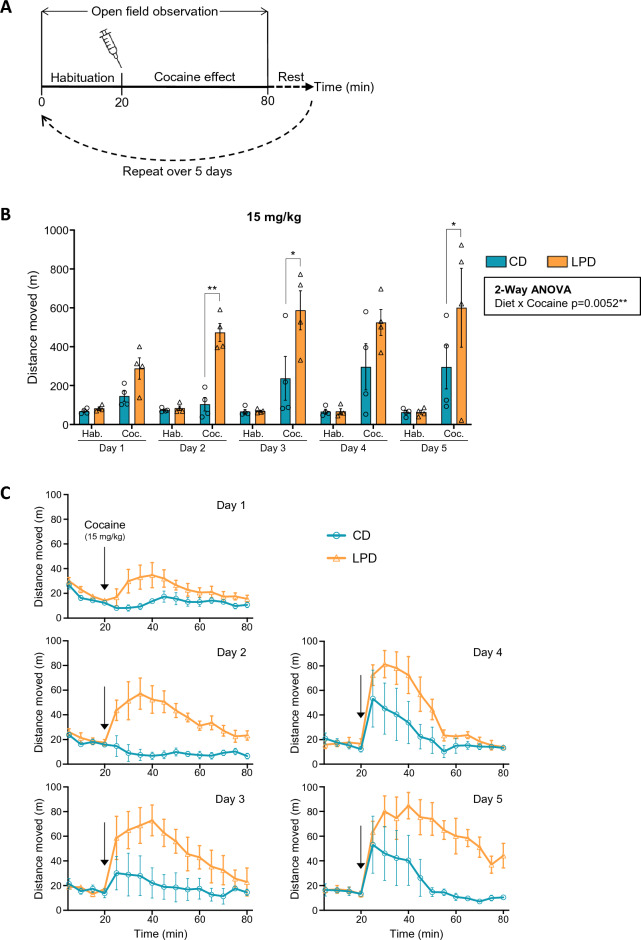
Increased cocaine sensitivity in offspring exposed to LPD in utero. (**A**) Diagram illustrating 5 day cocaine administration and observation regime. (**B**) Quantification of distance moved during a 5 day trial involving daily administration of 15 mg/kg cocaine, for CD- or LPD-gestationally exposed adult offspring. Two-way repeated measures ANOVA revealed a significant interaction between diet and cocaine (p = 0.0052), with Sidak’s multiple comparisons test revealing no differences during habituation on any days (padj > 0.05), but significantly increased activity in LPD mice post-cocaine on days 2 (**padj = 0.0058), 3 (*padj = 0.0104) and 5 (*padj = 0.0382). CD n = 4, LPD n = 4; error bars = SEM. (**C**) Time course comparisons (5 min intervals) of distance moved during habituation (first 20 min) and post-cocaine administration (60 min) in adult CD or LPD-exposed mice during 5 days of cocaine sensitisation, using 15 mg/kg cocaine. CD n = 4, LPD n = 4; error bars = SEM.

Dopaminergic cells in the midbrain are involved in movement regulation and pharmacological stimulation of the dopamine system results in increased extracellular dopamine concentration and hyperactivity in rodents^39^. Overexpression of *Cdkn1c* can alter dopamine circuitry in mice^20,27,29^ and has been associated with hyperactivity in humans^40^. To explore this, we examined the responses of CD- or LPD-exposed offspring to cocaine. Cocaine binds to the dopamine transporter (DAT) and thereby blocks the removal of extracellular dopamine at the synapse. Previous studies had indicated that cocaine elicits a heightened locomotor response in mice that had been exposed to protein restriction throughout gestation and lactation^27^. Adult male mice that had been exposed to LPD or CD in utero were injected daily with cocaine for 5 consecutive days (as shown schematically in Fig. 3A). This regime of administration enables cocaine sensitisation to be determined by analysing the impact of repeat challenge, relative to initial responses. During habituation, prior to drug exposure, both sets of adult mice showed broadly similar locomotor activity as depicted in Fig. S2A (left) and Supplementary Videos S1, S2. To examine behavioural sensitivity to cocaine, we initially administered 20 mg/kg and observed high levels of stereotypic circling behaviour (Supplementary Videos S3, S4, and Fig. S2A) making it difficult to accurately assess distances travelled by these animals (Fig. S2B, C), which can sometimes occur in response to high doses of psychostimulants^41^. We therefore administered a lower dose of cocaine (15 mg/kg) and in this case clearly observed cocaine-induced hyperactivity which was more pronounced in LPD animals compared to CD controls, indicating a heightened sensitivity to cocaine (Fig. 3B, 3C). Tight circling behaviour observed in LPD offspring exposed to higher doses of cocaine (20 mg/kg) has been reported by others using higher amphetamine and cocaine doses than administered here^41,42^. In addition, similar stereotypical behaviours in response to cocaine have been reported in the offspring of dams exposed to LPD for 5 weeks prior to mating and through pregnancy^43^ as well as in female rats maintained on a LPD diet^44^.

**Figure 4:**
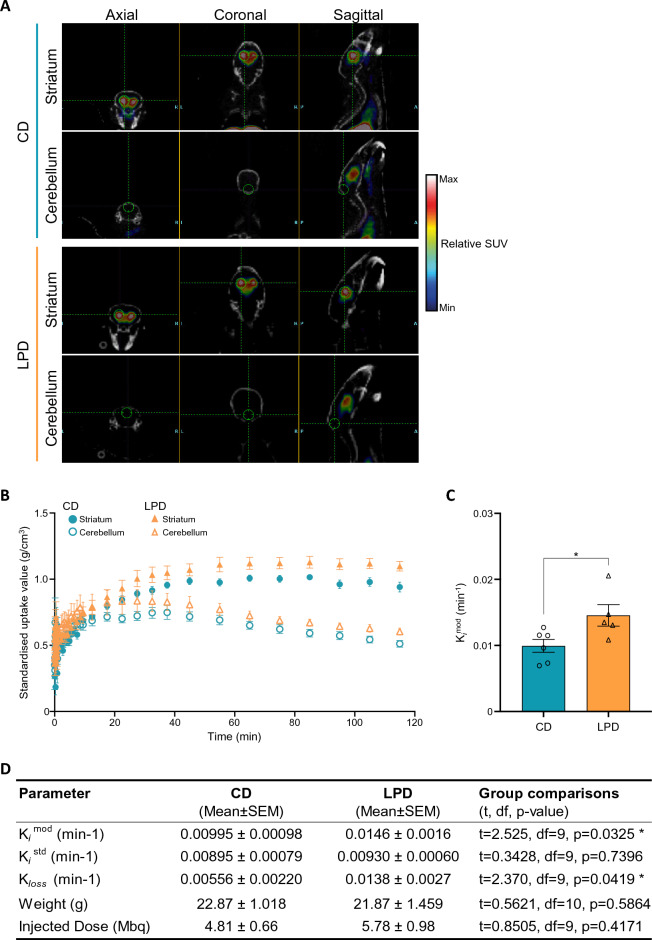
Altered striatal dopamine function in offspring exposed to LPD in utero, measured by PET imaging. (**A**) Representative PET images of axial, coronal and sagittal views of adult male CD- or LPD-exposed mouse brains showing regions of interest drawn around the striatum and cerebellum. (**B**) Time activity curves of mean striatal (filled shapes) and cerebellar (open shapes) [^18^F]FDOPA radioactivity signal in CD-exposed (circles) or LPD-exposed male mice (triangles), sampled during a 2 h PET scan. Radioactivity is presented as standardised uptake values (SUVs), corrected for mouse body weight, injected radiotracer dose and time of injection. CD n = 6, LPD n = 5; error bars = SEM. (**C**) Comparison of CD and LPD K_*i*_^mod^ values, a measure of striatal dopamine synthesis capacity which corrects for loss of radioactive metabolites from the striatum throughout the scan. Each data point represents an animal (CD n = 6, LPD n = 5, with two striatal values averaged per animal); error bars = SEM; two-tailed unpaired *t*-test (*p = 0.0325); large effect size (Cohen’s d = 1.495). (**D**) Summary of μPET Parameters. CD n = 6, LPD n = 5; two-tailed unpaired *t*-tests (*p < 0.05).

To ask whether this increased sensitivity reflects elevated dopamine synthesis capacity in LPD-exposed animals, we used micro-positron emission tomography (PET) imaging with the radiolabelled tracer [^18^F]FDOPA to examine 12 adult mice, six for each gestational exposure. [^18^F]FDOPA PET imaging has been widely used to interrogate dopamine synthesis capacity in a variety of neurological and neuropsychiatric disorders, and is a validated approach to assess nigrostriatal dopamine circuit integrity^45,46^, including in mice^47,48^. Briefly, PET and CT scans were aligned and 3-dimensional regions of interest drawn manually around the left and right striata, midbrain and cerebellum (which served as a control for non-specific uptake) (illustrated in Fig. 4A). Time activity curves were extracted from the data (Fig. 4B) and modelled by Gjedde-Patlak analysis^49^ to derive multiple measures of dopaminergic activity, including the rate constant, K_*i*_^mod^, for the striatal uptake of [^18^F]FDOPA and its conversion to [^18^F]fluorodopamine, corrected for dopamine turnover during the scan (Fig. 4C and Supplementary Fig. S3A, B, summarised in Fig. 4D). LPD-exposed mice showed a significant increase in K_*i*_^mod^ relative to controls (Fig. 4C), and K_*loss*_ (an index of dopamine turnover) was also significantly increased in these animals. K_*i*_^std^ values, which estimate dopamine synthesis capacity but do not correct for loss of radioactive metabolites over the 2 h period of data collection, were not significantly different between the groups, likely because of the higher K_*loss*_ in LPD-exposed mice. Together these data confirm that gestationally LPD-exposed mice show increased striatal dopamine synthesis capacity and dopamine turnover, indexed by [^18^F]FDOPA imaging, relative to animals gestationally exposed to control diet.

### Comparing gene expression in the brains of adult mice following in utero LPD or CD exposure

**Figure 5:**
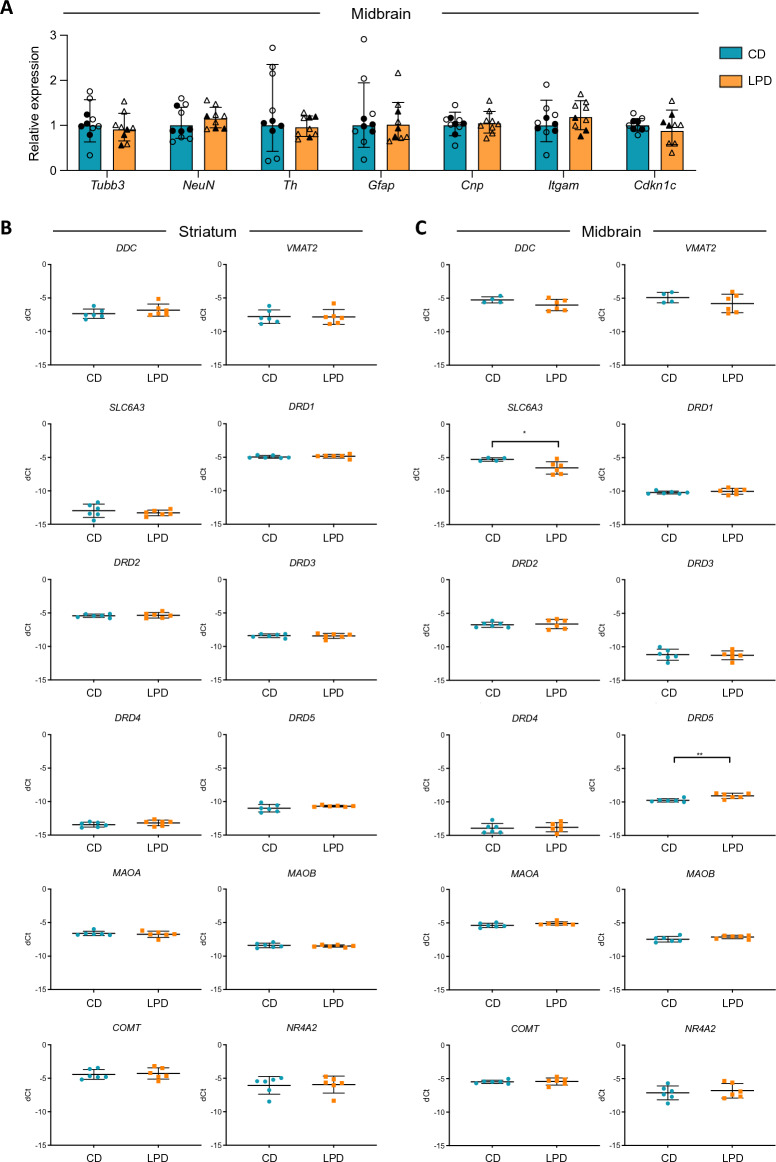
Comparison of gene expression in adult mouse brain of offspring exposed to CD or LPD during gestation. (**A**) Quantitative RT-PCR analysis of *Tubb3* (immature neurons), *NeuN* (mature neurons), *Th* (dopaminergic neurons), *Gfap* (astrocytes), *Cnp* (oligodendrocytes), *Itgam* (microglia) and *Cdkn1c* transcript expression in dissected adult (8–12 weeks old) midbrain of mice that had been exposed to LPD (orange) or CD (blue) in utero. Expression was normalised to *β-actin* and is plotted relative to CD. Results combine animals previously subjected to behavioural challenge (CD n = 6, LPD n = 6, open symbols) and those not previously exposed (CD n = 4, LPD n = 3, filled symbols). Bars show geometric mean; error bars = geometric SD; unpaired *t*-tests were used to compare CD with LPD, no significant differences were detected (p > 0.05). (**B**,**C**) Relative expression of genes encoding proteins that are particularly relevant to dopamine uptake and metabolism in adult (8–12 weeks old) mouse striatal (**B**) and midbrain samples (**C**) are shown. Expression is shown as average delta-CT relative to *β-actin.* Decreased expression of *SLC6A3,* encoding DAT (*p = 0.0298), and increased expression of *DRD5*, encoding dopamine receptor D5 (**p = 0.0050), was detected (two-tailed unpaired *t*-tests; n = 6 for each diet group; error bars = SD).

To better understand the biological basis of increased dopamine synthesis capacity in LPD animals, we examined the expression of a panel of genes that characterise different cell types within the adult brain using quantitative RT-PCR. As shown in Fig. 5A, expression of *Tubb3* and *NeuN* (which distinguish immature and mature neurons) was similar in the midbrain of LPD and CD-derived samples. Similarly, we saw no significant differences in expression of *Gfap, Cnp* and *Itgam,* markers of astrocytes, oligodendrocytes and microglia, respectively. Perhaps surprisingly, we did not see a significant increase in *Th* or *Cdkn1c* expression in LPD- as compared to CD-exposed midbrain samples (Fig. 5A). Furthermore, expression levels of these genes in LPD- and CD-exposed samples derived from other regions of the brain (cortex, cerebellum and striatum) were similar (Supplementary Fig. S4A–C), and were broadly equivalent in animals subjected to behavioural tests (depicted as open symbols) or naïve to testing (closed symbols, Fig. 5A). Previous studies in which maternal LPD exposure extends throughout breeding, pregnancy and lactation have reported a > sixfold elevation in *Th* expression in all regions of the brain examined, as well as upregulation of *DAT* (*SLC6A3*) and *DARPP-32*^27^. Here we show that exposure to LPD solely during gestation was, in contrast, insufficient to cause such sustained increases in *Th* expression, despite inducing increases in the number of TH-positive midbrain neurons. To investigate this further we analysed the expression of a panel of genes implicated in dopamine synthesis and metabolism, such as *DDC* (*Aadc*), *SLC6A3* (*DAT*), dopamine receptors *DRD1, DRD2, DRD3, DR4 and DRD5*, or genes implicated in interactions with *Cdkn1c*/p57^KIP2^, such as *NR4A2* (*Nurr1*), in both striatum and midbrain (Fig. 5B, C). We observed a very modest increase in *DRD5* expression in LPD-derived midbrain, together with a slightly reduced expression of *SLC6A3* (*DAT*). Overall, significant changes in the expression of key neuronal- or dopamine-associated genes were not detected. By normalising gene expression to different housekeeping controls (for example *18S*, *Tbp* and *Gapdh*, rather than *β-actin*), small differences in gene expression between LPD and CD samples were in some cases statistically significant (as shown for *Cdkn1c* in Supplementary Fig. S4D left; *DARPP-32* expression was unaffected by this, as shown in Supplementary Fig. S4D right). In addition, we noted that *SLC6A3* (*DAT*) and DRD5 expression were not significantly altered by dietary exposure in a separate cohort of animals (Supplementary Fig. S4E). In conclusion, the scale of gene expression changes detected herein appear vanishingly low in comparison with those previously reported, where offspring were exposed to maternal LPD for much longer periods of time, notably throughout gestation, early prenatal life and lactation^27^*.*

## Discussion

Numerous studies in the last few decades have examined the impact of early life adversity on offspring health and the possible mechanisms that underlie an increased susceptibility to neuropsychiatric and metabolic disorders. In rat models, protein restriction during pregnancy has been shown to result in elevated levels of dopamine in the brain of offspring^50,51^, increased TH activity^50^ and altered dopamine receptor binding^52^. Similarly, studies in mice have shown a sustained overexpression of *Th* (six to eightfold) in the ventral tegmental area (VTA), nucleus accumbens, prefrontal cortex and hypothalamus of adult mice that were exposed to LPD throughout prenatal development and postnatally until weaning^27^. Here we show that in offspring exposed to LPD only during gestation, there are much more modest changes in gene expression and the levels of most neuronal or dopamine-associated genes tested remained relatively unchanged. In light of prior studies, these data were unanticipated. It is possible that differences in the scale of gene expression changes herein, compared with those described previously^27^, reflect differences in mouse husbandry, genetic background, or microbiomes, but perhaps more likely, reflect the different durations of dietary exposure. Susceptibility to dietary challenge changes during mouse ontogeny; since the timing of LPD exposure was different between these studies, longer exposure incorporating lactation (as in^27^) may serve to stabilize or even increase epigenetic changes in gene activity. In this regard, we have previously shown that female mice injected with chromatin modifying drugs such as 5ʹazacytidine or Trichostatin A mid-way through pregnancy displayed overt paternal *Cdkn1c* de-repression in embryos, but this was transient and not retained postnatally^28^. In contrast, dietary exposure to LPD or to a high fat diet (HFD) throughout pregnancy has been shown to provoke more long-lasting epigenetic changes, exemplified by a sustained loss of *Cdkn1c* or *Dlk1-Dio3* imprinting respectively, that persists long after dietary challenge is withdrawn^28,53^. Following in utero HFD exposure we have shown altered phenotypes among immediate offspring, as well as their progeny, illustrating a surprising durability of some exposure-induced epigenetic change^53^. It is conceivable that extended maternal exposure to LPD after birth and during lactation, a period of development in the mouse that correlates with the final trimester of human foetal development, serves to heighten or reinforce changes in gene expression initiated much earlier in development.

Whatever the explanation, we have shown here that exposure to LPD solely in utero prompts elevated dopamine synthesis capacity judged by micro-PET imaging. These animals have increased numbers of TH-positive cells and display behaviours consistent with altered dopamine circuits in adolescent and adult animals. While it is difficult to unequivocally prove that LPD-induced loss of *Cdkn1c* imprinting drives these outcomes, several pieces of evidence now implicate a causal role for *Cdkn1c*/p57^KIP2^. Firstly, ourselves and others have shown that protein deprivation in pregnancy results in an erosion of DNA methylation across the sDMR in embryonic brain, with inappropriate re-expression of paternal *Cdkn1c* by cells already expressing maternally-derived *Cdkn1c*^27,28^. Supplementation of maternal LPD with folate as a source of methyl-donors, corrects methylation across the sDMR and substantially reduces *Cdkn1c* misexpression in the resulting offspring^28^. These results show some similarity to prior genetic experiments where relatively mild over-expression of *Cdkn1c* was shown to be sufficient to provoke increased numbers of TH-positive cells in the periventricular hypothalamus and VTA, and a range of behavioural abnormalities that included altered reward-related and social dominance behaviours^20–22,29^. Maternally inherited loss of function of Cdkn1c has also been reported to result in reduced numbers of Nurr1-positive and TH-positive cells in the ventral midbrain at E18.5^54^. Taken together, these studies provide formal proof that changes in TH-positive neuron number are due to *Cdkn1c* dosage.

While the precise molecular and cellular mechanisms that link in utero LPD exposure to increased TH-positive cell numbers in the midbrain of adult offspring are not fully understood, we know that erosion of DNA methylation at the paternally-inherited somatic DMR of *Cdkn1c* occurs in response to gestational LPD, and results in overexpression in the developing embryo^28^. Accumulating evidence suggests that Cdkn1c is required for maturation of midbrain dopamine neuronal cells and that this may not require the cell cycle inhibitory role of Cdkn1c, but instead requires a protein–protein interaction with Nurr1^54^. This suggests that elevated Cdkn1c expression may increase the number of TH-positive cells at least in part by promoting their differentiation and preventing apoptosis. In this regard, low level *Cdkn1c* expression has been reported to restrain apoptosis in the neocortex^25^, and interactions between *Cdkn1c*/p57^KIP2^ and Nurr1 can promote postmitotic differentiation of dopamine neurons ^54,55^ and may influence oxidative stress and survival^56–58^. This suggests that LPD-induced paternal *Cdkn1c* de-repression may increase neuron number, both by cell cycle-dependent and cycle-independent mechanisms.

The epigenetic mechanisms that link *Cdkn1c* LOI to gestational exposure to LPD are not fully understood although there is compelling evidence showing that DNA methylation at the somatic DMR is required to maintain paternal *Cdkn1c* silencing and loss of DNMT1 results in *Cdkn1c* LOI^59,60^. Consistent with a major role for DNA methylation in maintaining paternal *Cdkn1c* silencing, LPD-induced erosion of somatic DMR methylation is rescued by dietary supplementation with folate^28^. Other epigenetic mechanisms have been implicated in regulating imprinting, for example tri-methylation of histone H3 at lysine 27 (H3K27me3), catalysed by polycomb repressor complex 2 (PRC2)^61–65^. In addition, we have recently shown that inhibitors that block DNMT1, HDACs or BET activity can also partially relieve paternal *Cdkn1c* silencing in cultured cells derived from *Cdkn1c-Fluc-lacZ* reporter mice^66^.

Altered TH-positive cell number, dopamine circuitry and changes in offspring behaviour are consistent features described herein and in other studies of protein restriction in pregnancy. Our data align with results showing that BAC-*Cdkn1c* transgenic mice are hypersensitive to amphetamine and display changes in behaviour that are routed in the mesolimbic dopaminergic system^20^ and underscore the potential of maternal nutrition to transform the long-term physiology and neurobehavioral outcomes of offspring. The observation that in utero exposure to LPD induces an increased number of dopamine neurons in offspring, coupled with increased locomotor activity, reduced anxiety and a greater propensity to explore, is intriguing, particularly as this phenotype could offer distinct survival advantages for animals born into a nutritionally-deprived environment. In this setting prenatal epigenetic modifications that are environmentally induced, such as paternal *Cdkn1c* de-repression, would remain intrinsically reversible but could alter postnatal behaviour to enhance offspring survival, without requiring the genome to be changed or compromised. Interestingly, poor rotarod performances of juvenile LPD offspring improved as animals matured, alongside elevated capacity in open field, elevated O-maze and marble burying tests. This suggests that potential motor functional deficits in LPD offspring can be normalised in adulthood, despite an increase in TH-positive cells in the midbrain. On the other hand, LPD-exposed offspring displayed behavioural alterations that appeared much more long-lasting, such as hyperactivity, reduced levels of anxiety and altered responses to cocaine, that most likely are a consequence of remodelling the dopaminergic system in development in response to environmental challenge.

These demonstrations raise concerns about similar potential vulnerabilities during human pregnancy. Changes in dopamine levels and circuitry are linked to perturbations in reward processing, threat responses and hyperactivity, as well as increasing the risk of neuropsychiatric impairments^67^. Elevated dopamine synthesis capacity has, for example, been observed in the striata of people at risk of or who have schizophrenia^68,69^ and in the prefrontal cortex of people with attention deficit hyperactivity disorder (ADHD)^70^. Preclinical models that allow epigenetic changes to be longitudinally tracked for the first time in living animals^28,53^, such as the *Cdkn1c-Fluc-lacZ* reporter line described herein, enable the impacts of environmental challenges to be visualised, monitored and potentially mitigated, from in utero sensitivity through perinatal development, adolescence, adulthood and ultimately across generations. In conjunction with detailed epidemiologic data, these models can offer an appealing route to systematically uncover the mechanisms and aetiology that link prenatal adversity with later life outcomes, and test protective strategies for ameliorating the negative impacts of such exposures.

## Materials and methods

### Animals

All animal procedures were undertaken in accordance with the UK animals (scientific procedures) Act 1986, were approved by the Imperial College AWERB committee, and were performed under a UK home office project license. Findings and experiments described in this paper were designed and reported following the Animal Research: Reporting of In Vivo Experiments (ARRIVE) guidelines.

The *Cdkn1c-Fluc-lacZ* reporter mouse line was genetically engineered by Taconic Biosciences and mice were genotyped as described previously^28^. All mice were maintained on a 129S2/SvHsd background. Mice were housed groups of 4–6 in pathogen-free barrier facilities, and maintained under a controlled environment (12 h light/dark cycle, 21 + / − 2 ℃ and 45–65% humidity) with food and water ad libitum. The cage environment was enriched with Aspen bedding, tissues for nesting, wooden chew blocks and tunnels. In the breeding cages the tunnels were replaced with cardboard mouse houses. For timed matings, 2–6 month-old males were set up with up to three females of a similar age with daily inspections for vaginal plug. Females were removed from the cage upon vaginal plug discovery, which was considered E0.5 for embryonic development.

### Diet

Mice were given regular rat and mouse Nr.3 (RM3) breeding chow (801700, 22.45% protein, 2.99 ppm folic acid, Special Diet Services) for general breeding purposes. For the in utero dietary exposure experiments, after discovery of a vaginal plug, females were randomly assigned either low protein diet (8.5% protein purified diet, 4.1 ppm folic acid, 5769, TestDiet) or a calorie-matched control diet (18.6% protein basal diet, 5755, TestDiet) and kept on that diet until birth, when animals were returned to a regular chow diet.

### Animal transfers

Mice were transferred to designated imaging suites for bioluminescent imaging and PET scanning in transport boxes with tissue and food from the original cage to reduce stress and make the transfer more familiar. Transfers were performed at least 30 min before the start of the bioluminescent imaging experiments, and at least 2 h before the start of the PET scans, to ensure the animals were habituated to the new environment. For behavioural experiments, mice were transferred to a different animal facility at least a week before the first behavioural test.

### Bioluminescent imaging

Adult mice were injected (intraperitoneal (IP) injection) with D-luciferin (D-luc) (Perkin Elmer) at a dose of 0.15 mg/g and then anaesthetised with isoflurane. 10 min after D-luc injection mice were imaged on an IVIS spectrum (Perkin Elmer) using 180 s exposure, Bin 8, and FOV C or D depending on the number of mice. Images were acquired and analysed on the Living Image software (Perkin Elmer, version 4.7.3). For quantification of bioluminescent signal, identical surface area regions of interest (ROIs) were drawn around the subjects and total flux (p/s) was plotted. Pregnant dams were imaged 5 min after D-luc injection and then culled by cervical dislocation. Embryos were dissected and placed in multi-well plates for bioluminescent imaging. The embryos were imaged for 60 s, FOV A-C, Bin 4, focus 1 cm. No additional D-luc was used for embryonic imaging.

### Micro-PET imaging

[^18^F]FDOPA was produced by Invicro (London, UK) as previously described^71^ in a subatomic particle accelerator, the cyclotron. [^18^F]FDOPA had to be over 95% pure to be used for in vivo applications. Male 8–12 week-old mice were allowed to habituate for at least 2 h in the imaging facility before the scanning procedure. Mice were anaesthetised with 5% isoflurane, underwent external jugular vein cannulation and were maintained under terminal 1.5–2.5% isoflurane anaesthesia. Mice were injected IP with the COMT and AADC inhibitors entacapone (40 mg/kg) and benserazide hydrochloride (10 mg/kg) 45 min and 30 min respectively, prior to the [^18^F]FDOPA scan to reduce peripheral radiotracer uptake and improve uptake in the brain^49^. In total, 12 male mice were scanned (six for each diet). One LPD mouse was subsequently excluded because the scan data was not of sufficient quality to be analysed/quantified.

Scans were acquired with a Siemens Inveon µCT/PET scanner. A 20 min CT scan was performed prior to the injection of the radiotracer for attenuation correction. A dynamic PET scan was performed directly after intravenous administration of a minimum of 2 MBq [^18^F]FDOPA in the external jugular vein. Emission data was acquired for 2 h and split into 43 frames with increasing time duration. At the end of the scan, the subject was quickly removed from the CT/PET scanner and culled via cervical dislocation. Throughout the scanning period, the body temperature of the subject was monitored with a rectal thermometer and maintained at 37 ℃with the help of a heating lamp when necessary. The respiration rate of the animal was monitored using the BioVet software (BioVet software; m2m Imaging Corp, Cleveland, OH, USA), and the isoflurane percentage was adjusted between 1.5–2.5% to maintain a steady breathing rate. Radioactivity of [^18^F]FDOPA was measured before dosing. Leftover radioactivity (post-dose) as well as radioactivity lost on gloves and used syringe (waste) was also measured and accounted for when calculating the injected dose.

### PET image analysis

Images acquired from PET scan were analysed with the Inveon Research Workplace software. The CT and PET scan images were aligned and 3D ROIs were drawn manually around the right and left striata (0.07 cm^3^), as well as the cerebellum (0.1 cm^3^). The cerebellum was used as a reference region to account for non-specific uptake of [^18^F]FDOPA, given the insignificant dopaminergic projections^49^. Time-activity curves were extracted from the PET data and modelled by Gjedde–Patlak analysis to derive multiple measures of dopamine synthesis capacity^49,72^. K_*i*_^std^ represents the influx rate constant of the uptake and conversion of [^18^F]FDOPA to [^18^F]fluorodopamine in the striatum relative to the cerebellum, using the scan data acquired between 10 and 60 min^49^. The other measure of dopamine synthesis capacity, K_*i*_^mod^, was calculated through an extended Gjedde-Patlak analysis that uses the radioactivity profile from 20 to 90 min after tracer administration adjusted for the catabolism of [^18^F]fluorodopamine during the course of the scan^49^. This analysis also derived an estimate of the rate constant for the breakdown of [^18^F]fluorodopamine to its metabolites, termed K_*loss*_*,* representing turnover of dopamine^49,73^. Data were analysed after all scans were completed.

### Behavioural testing

All behavioural tests were carried out during daytime and were carried out in a dimly lit room, free from visual and auditory disruptions, to ensure similar conditions for each animal. Both males and females were used for behavioural testing. Two distinct cohorts of mice were examined, representing mice at juvenile (4–5 weeks) and adult (9–10 weeks) mice. Animals were allowed to habituate for at least 10 min in the behaviour room prior to testing, and no more than eight animals were kept in the room at one time. 9 week-old females were not exposed to cocaine; otherwise all animals underwent all behavioural tests unless they showed signs of sickness or were found dead (two mice in total out of 104), in the same order (open field, rotarod, elevated O-maze, marble burying, Y-maze and cocaine sensitisation), with a 1–2 day break between experiments. Experimenters were blinded to group identity during data collection and early stages of analysis for all behaviour experiments.

### Open field testing

Mice were habituated in the designated room for 10 min before the start of the experiment. They were then placed in the centre of individual open-field arenas (45 cm^3^) filled with 1 cm of sawdust, and assessed for baseline locomotor activity in batches of four. Activity was recorded for 60 min on the EthoVision XT tracking system (Noldus Information Technologies, Leesburg, VA, USA). Total distance travelled and speed of movement for each mouse were calculated in 5 min bins as well as hour-long totals.

### Rotarod

Mice were trained on a rotarod (47600, Ugo Basile, Italy) using a protocol of three trials per day with an inter-trial interval of 1 h for three consecutive days. The rotarod accelerated from 5 to 60 rpm over a period of 10 min. Latency to fall and rod speed at the moment of fall were recorded for each mouse. Average latency to fall per trial and per day were calculated.

### Y-maze

Mice were tested in an opaque Y-shaped maze with three arms positioned at 120° from each other. Mice were individually placed at the end of an arm facing away from the centre of the maze, alternating between starting arms with each mouse to account for any bias. Arm exploration was observed for 5 min, and spontaneous alternation (ABC pattern), alternate arm entries (ABA pattern) and same arm entries (ABB pattern) were recorded. Speed of movement and total distance travelled were also recorded with the EthoVision XT video tracking system.

### Elevated O-maze

A 5.5 cm wide ring-shaped runway with an outer diameter of 46 cm was placed 40 cm above the floor. Two opposing 90° sectors were protected by 16 cm high inner and outer white walls (closed sectors). The remaining two 90° sectors were without walls (open sectors). Animals were released at the edge of one of the closed arms facing inwards and observed for 5 min. The EthoVision XT video tracking system was used to record time each mouse spent on open and closed arms and number of entries in open and closed sectors. An entry was defined as an animal entering that specific sector with all three body parts (head, centre and base of tail).

### Marble burying

Mice were put in an open field arena filled with 10 cm of sawdust, where 20 glass marbles had been previously overlaid in a 4 × 5 arrangement, and were tested for 20 min in batches of four and after removing the mice, the coverage of marbles was scored. The intensity of behaviour was scored by counting the buried marbles at the end of the task. Marbles were considered buried if they were more than 2/3 covered. The results were confirmed by a second observer; there were no disagreements.

### Cocaine challenge

Cocaine sensitisation experiments were carried out over five successive days (starting at 10am), treating four animals simultaneously. Each day, mice were weighed and then allowed to habituate for 20 min in the open field. After 20 min, they were each given IP injections of cocaine (Sigma–Aldrich, C5776) at 20 mg/kg or 15 mg/kg, in a solution of 4 mg/ml. Their horizontal locomotor activity was recorded in the open field for 60 min with EthoVision XT. Two LPD-exposed mice administered with 20 mg/kg cocaine exhibiting outlier locomotor behaviour were excluded from the analysis.

### Molecular analysis of gene expression

Mouse tissues were dissected on ice^74^, flash frozen in liquid nitrogen, and stored at − 80 ℃. For RNA extraction, samples were transferred on dry ice, homogenised on the TissueLyserII (Qiagen, 85300), with the addition of a stainless-steel bead (5 mm, Qiagen, 69989) and then were processed with the AllPrep DNA/RNA mini kit (Qiagen, 80204). DNase treatment (RNase-free DNase kit, Qiagen, 79254) was incorporated in the RNA extraction process. To produce cDNA, 500 ng of RNA was used in a reverse transcription reaction, using random primers and SuperScript III (SuperScript^™^ III reverse transcriptase, Thermo Fisher, 18080085) according to the manufacturer’s instructions. The cDNA was diluted 1:5 for quantification by qPCR, using 2 µl of cDNA per reaction and two technical replicates per sample. Relative gene expression was calculated by normalising to ubiquitously expressed control genes (including *β-actin*, *Tbp, 18S* and *Gapdh*), as indicated in the figure legends. The primers used are shown in Table 1.

**Table 1:** Primers used in qRT-PCR analysis.

Target	Forward primer sequence	Reverse primer sequence
*Tubb3*	TAGACCCCAGCGGCAACTAT	GTTCCAGGTTCCAAGTCCACC
*NeuN*	GTAGAGGGACGGAAAATTGAGG	GTGGGGTAGGGGAAACTGG
*Th*	CCAAGGTTCATTGGACGGC	CTCTCCTCGAATACCACAGCC
*Gfap*	CGGAGACGCATCACCTCTG	AGGGAGTGGAGGAGTCATTCG
*Cnp*	TTTACCCGCAAAAGCCACACA	CACCGTGTCCTCATCTTGAAG
*Itgam*	CCATGACCTTCCAAGAGAATGC	ACCGGCTTGTGCTGTAGTC
*Cdkn1c*	AGAGAACTGCGCAGGAGAAC	TCTGGCCGTTAGCCTCTAAA
*DDC (Aadc)*	TAGCTGACTATCTGGATGGCAT	GTCCTCGTATGTTTCTGGCTC
*VMAT2*	ATGCTGCTCACCGTCGTAG	GGACAGTCGTGTTGGTCACAG
*SLC6A3 (DAT)*	AAATGCTCCGTGGGACCAATG	GTCTCCCGCTCTTGAACCTC
*DRD1*	ATGGCTCCTAACACTTCTACCA	GGGTATTCCCTAAGAGAGTGGAC
*DRD2*	ACCTGTCCTGGTACGATGATG	GCATGGCATAGTAGTTGTAGTGG
*DRD3*	CCTCTGAGCCAGATAAGCAGC	AGACCGTTGCCAAAGATGATG
*DRD4*	GCCTGGAGAACCGAGACTATG	CGGCTGTGAAGTTTGGTGTG
*DRD5*	CTCGGCAACGTCCTAGTGTG	AATGCCACGAAGAGGTCTGAG
*MAOA*	GCCCAGTATCACAGGCCAC	CGGGCTTCCAGAACCAAGA
*MAOB*	ATGAGCAACAAAAGCGATGTGA	TCCTAATTGTGTAAGTCCTGCCT
*COMT*	CTGGGGGTTGGTGGCTATTG	CCCACTCCTTCTCTGAGCAG
*NR4A2 (Nurr1)*	GTGTTCAGGCGCAGTATGG	TGGCAGTAATTTCAGTGTTGGT
*DARPP-32*	CCCATCACTGAAAGCTGTGC	TCCCGAAGCTCCCCTAACTC
*β-actin*	CATCCGTAAAGACCTCTATGCCAAC	ATGGAGCCACCGATCCACA
*18S*	GTAACCCGTTGAACCCCATT	CCATCCAATCGGTAGTAGCG
*Tbp*	GAAGAACAATCCAGACTAGCAGCA	CCTTATAGGGAACTTCACATCACAG
*Gapdh*	AAGAGAGGCCCTATCCCAACTC	TTGTGGGTGCAGCGAACTTTATTG

### Tissue fixation, sectioning and immunofluorescence.

Mice were culled by cervical dislocation. Brains were immediately dissected on ice before fixation in 4% paraformaldehyde at 4 °C with gentle shaking for 48–72 h. Tissue was washed in PBS, transferred to a 30% sucrose solution, incubated in an OCT:30% sucrose solution (1:1 ratio) for 1 h at RT, before being frozen in OCT over dry ice and stored for later use at − 80 ℃. After calibration in the Leica CM1800 cryostat (Leica Microsystems) chamber for 20 min, tissue was sectioned at − 22 ℃ to − 18 ℃ to generate 35 µm sections.

For immunofluorescence, sections were gently transferred into 10% Normal Goat Serum (NGS) blocking buffer for 1 h at RT, incubated in 0.2% phosphate-buffered serum with 0.1% TritonX (PBSTx) with 2% NGS with TH antibody (Abcam ab76442, 1:1000) overnight at 4 ℃ with shaking. The next day, sections were washed in PBS three times for 10 min, and then incubated for 1 h 45 min in 0.2% PBSTx with 2% NGS and secondary antibody (Invitrogen A-11039, Alexa 488, goat anti-chicken, 1:1000). Sections were washed in PBS twice for 10 min, incubated with DAPI for 30 min and washed again in PBS twice for 10 min. The sections were then floated in a larger basin with PBS and mounted on negatively charged slides, sealed with Vectashield, and left overnight in the dark before being examined.

### Confocal microscopy and image analysis

Images were acquired on a Leica SP8 microscope. Three slides were simultaneously imaged on a 3-slide holder to streamline the imaging process. Images of whole slides were initially acquired at × 2.5 magnification at low resolution (256 × 256) to locate the sections. Images of each section were then acquired with a 5 × objective at a higher resolution (1024 × 1024) to identify regions with TH signal. Lastly, tile scans of the substantia nigra (SN) and VTA were acquired with a 20 × objective at medium resolution (512 × 512), acquiring Z-stacks at 2 µm intervals through the entire tissue section. Sections spanning the VTA and SN (approximately between Bregma − 3.34 and − 3.66, based on reference to the Allen Mouse Brain Atlas, http://mouse.brain-map.org/) were used for cell body counts, using the central 20 µm of the acquired Z-stack. TH+ neurons were identified and counted in 3D using the Imaris software, with manual curation to correct for any mislabelled cells. At least two tissue sections were imaged and quantified per animal, and a minimum three animals were scored per condition. Both females and males were used for dopaminergic midbrain neuron counts. Representative images of the SN and VTA were processed in Fiji/Image J.

### Statistical analysis

All statistical tests were executed in GraphPad Prism (version 10.0.2) with details provided in the figure legends. No statistical methods were used to predetermine sample sizes. The behaviour data for each diet group passed the D’Agostino–Pearson normality test. No interaction was observed between sex and diet when analysing data from each timepoint separately, and therefore males and females were combined for all behaviour tests. For PET scans, Cohen’s d effect size was calculated using an online calculator (https://www.socscistatistics.com/effectsize/default3.aspx).

### Supplementary Information

Supplementary Figures.

Supplementary Video S1.

Supplementary Video S2.

Supplementary Video S3.

Supplementary Video S4.

## Data Availability

All data generated during this study are included in the article and its supplementary files or are available from the corresponding author on reasonable request.
