# Supplementary material for: Protein restriction during pregnancy alters Cdkn1c silencing, dopamine circuitry and offspring behaviour without changing expression of key neuronal marker genes
Source: Sci Rep. 2024 Apr 12;14:8528. doi: 10.1038/s41598-024-59083-7 (PMC11014953; doi:10.1038/s41598-024-59083-7)
Supplement: Supplementary file 1 — Supplementary Figures. [file 41598_2024_59083_MOESM1_ESM.pdf]

Supplementary Information

Protein restriction during pregnancy alters *Cdkn1c* silencing, dopamine circuitry and offspring behaviour without changing expression of key neuronal marker genes

Prodani, C. *et al.*

Supplementary Figures S1-S4

Legends for Supplementary Videos S1-S4

Figure S1

A

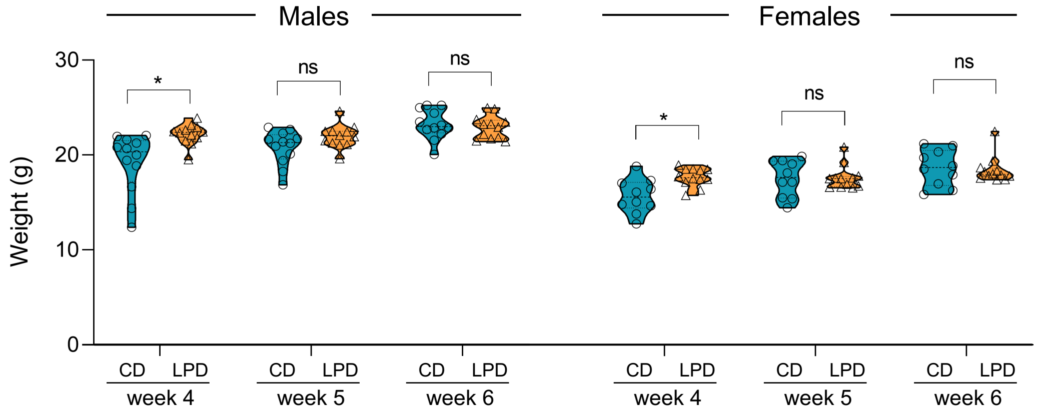

B

Elevated O-Maze

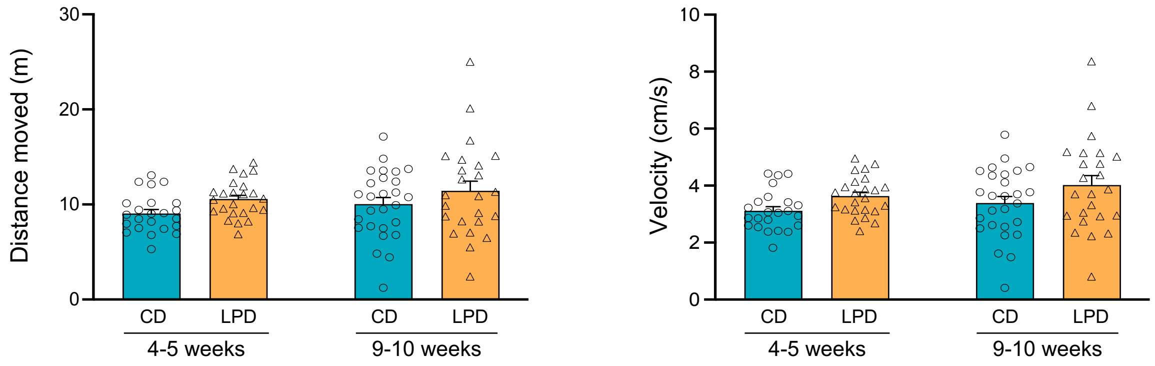

Supplementary Figure S1 | LPD-exposure in pregnancy impacts juvenile offspring weight.

Corresponding to Figure 2.

(A) Animals exposed to maternal CD or LPD in gestation were weighed at 4, 5 and 6 weeks of age. At 4 weeks, male (left) and female (right) LPD-exposed offspring (orange) weighed significantly more than CD-exposed (blue) offspring, but these differences normalised with age. Two-way repeated measure ANOVAs: Males (Age x Diet $p<0.0001$, Age $p<0.0001$, Diet $p=0.0721$, Subject $p<0.0001$), Females (Age x Diet $p<0.0001$, Age $p<0.0001$, Diet $p=0.3405$, Subject $p<0.0001$); Sidak's multiple comparisons tests (Males *padj=0.0228, Females *padj=0.0223); Males n=12, Females n=10; violin plots show median and quartiles.

(B) Analysis of distance moved (left) and velocity (right) during elevated O-maze testing. Diet had a significant effect both on distance moved (Two-way ANOVA: Diet $p=0.0343$, Age $p=0.1782$, Interaction $p=0.9344$) and on velocity (Two-way ANOVA: Diet $p=0.0144$, Age $p=0.1553$, Interaction $p=0.7939$). However, Sidak's multiple comparisons tests did not reveal any significant pairwise differences between diet groups or age groups (padj>0.05; 2 families of 2 comparisons per analysis). CD 4-5 weeks n=23, LPD 4-5 weeks n=24, CD 9-10 weeks n=27, LPD 9-10 weeks n=24; error bars=SEM.

Figure S2

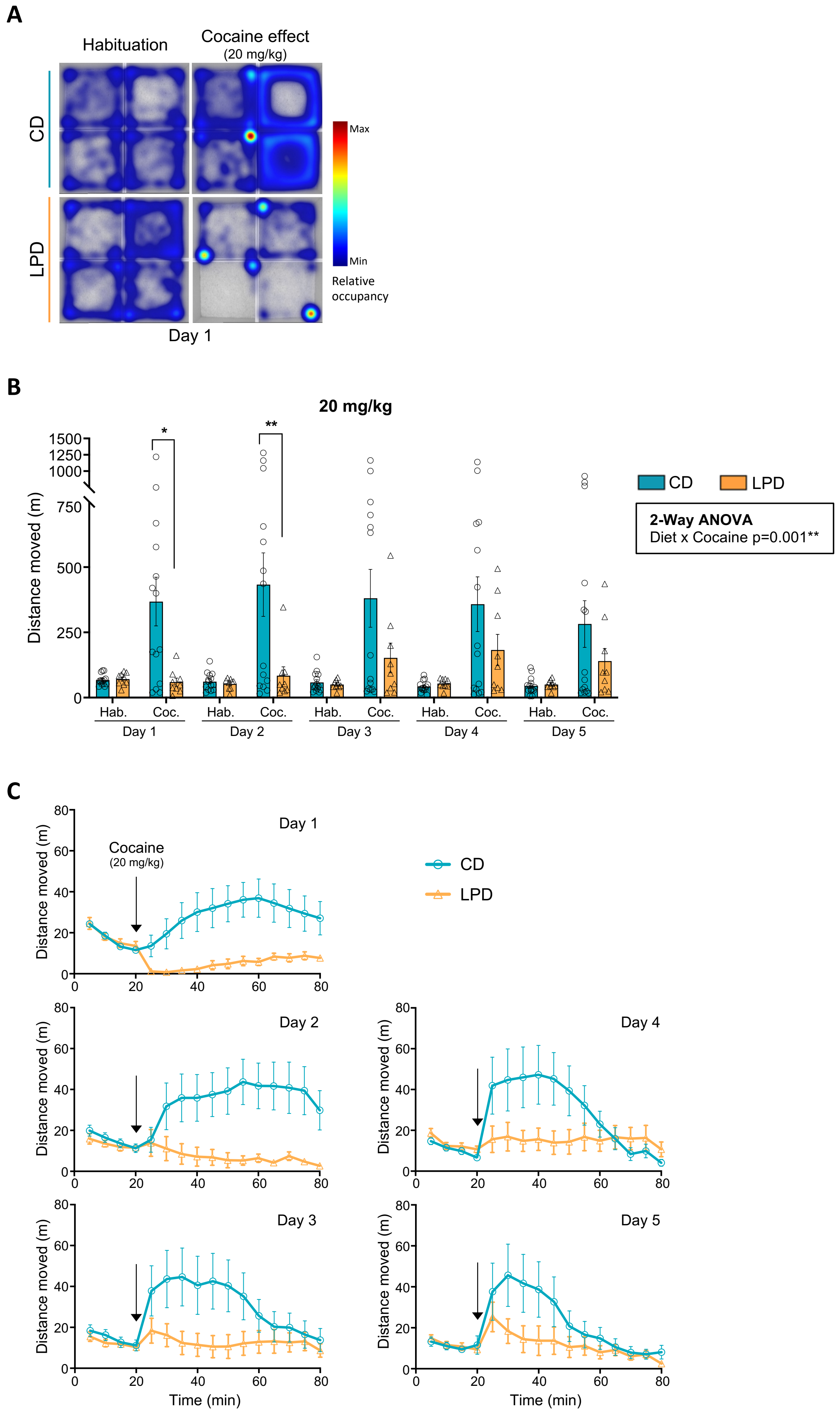
A
Habituation
Cocaine effect
(20 mg/kg)
CD
LPD
Max
Min
Relative occupancy
Day 1
B
20 mg/kg
Distance moved (m)
CD
LPD
2-Way ANOVA
Diet x Cocaine p=0.001**
Hab.
Coc.
Day 1
Day 2
Day 3
Day 4
Day 5
C
Cocaine
(20 mg/kg)
CD
LPD
Distance moved (m)
Time (min)
Day 1
Day 2
Day 3
Day 4
Day 5

Supplementary Figure S2 | Locomotor activity in response to cocaine administration.

Corresponding to Figure 3.

(A) Representative occupancy heatmaps showing locomotor activity of 4 CD- and 4 LPD-exposed adult mice before and after cocaine injection (20 mg/kg) on Day 1. For each treatment group, the movement of each mouse is summarised in an individual square.

(B) Quantification of distance moved during a 5-day trial involving daily administration of 20 mg/kg cocaine, for CD- (blue) or LPD- (orange) gestationally exposed adult offspring. Two-way repeated measures ANOVA revealed a significant interaction between diet and cocaine over the 5-day regime ($p=0.0010$). Sidak's multiple comparisons test confirmed no differences during habituation on any days ($p_{adj}>0.05$) but revealed significantly decreased activity in LPD mice post-cocaine on days 1 and 2 (**$p_{adj}=0.0039$, *$p_{adj}=0.0165$; 10 comparisons in total). CD n=14, LPD n=9; error bars=SEM.

(C) Time course comparisons (5 min intervals) of distance moved during habituation (first 20 min) and post-cocaine administration (60 min) in adult CD or LPD-exposed mice during 5 days of cocaine sensitisation, using 20 mg/kg cocaine. CD n=14, LPD n=9; error bars=SEM.

Figure S3

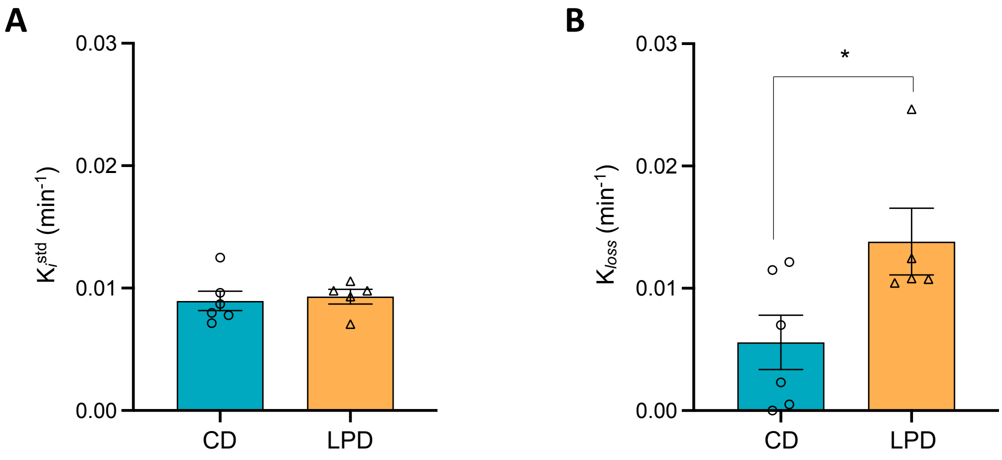

A
0.03
0.02
0.01
0.00
K_i^std (min^-1)
CD
LPD
B
0.03
0.02
0.01
0.00
K_loss (min^-1)
*
CD
LPD

Supplementary Figure S3 | Dopamine turnover is altered in the brains of LPD-exposed offspring.

Corresponding to Figure 4.

(A) Comparison of CD and LPD K_i^{std} values, a measure of dopamine synthesis capacity that omits the correction for loss of radioactive metabolites. Each data point represents an animal (CD n=6, LPD n=5, with striatal values averaged per animal); error bars=SEM; two-tailed unpaired t-test, p=0.7396.

(B) Comparison of CD and LPD K_{loss} values, an index of dopamine turnover. Each data point represents an animal (CD n=6, LPD n=5, with striatal values averaged per animal); error bars=SEM; two-tailed unpaired t-test (*p=0.0419).

Figure S4

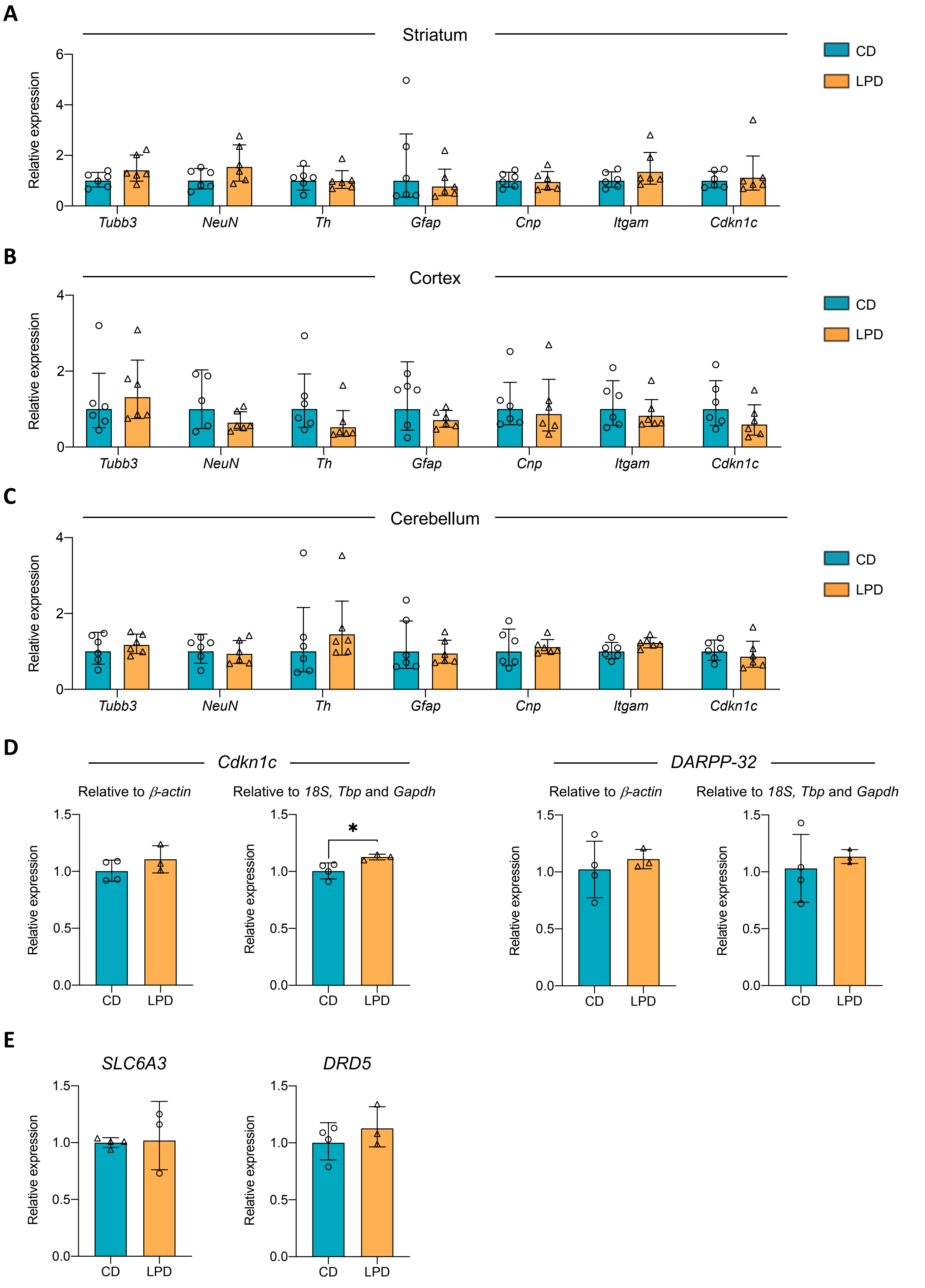
A
Striatum
CD
LPD
Relative expression
6
4
2
0
Tubb3
NeuN
Th
Gfap
Cnp
Itgam
Cdkn1c
B
Cortex
CD
LPD
Relative expression
4
2
0
Tubb3
NeuN
Th
Gfap
Cnp
Itgam
Cdkn1c
C
Cerebellum
CD
LPD
Relative expression
4
2
0
Tubb3
NeuN
Th
Gfap
Cnp
Itgam
Cdkn1c
D
Cdkn1c
DARPP-32
Relative to β-actin
Relative to 18S, Tbp and Gapdh
Relative to β-actin
Relative to 18S, Tbp and Gapdh
Relative expression
1.5
1.0
0.5
0.0
CD
LPD
*
E
SLC6A3
DRD5
Relative expression
1.5
1.0
0.5
0.0
CD
LPD

Supplementary Figure S4 | Gene expression in adult brain of *in utero* LPD- or CD-exposed mice.

Corresponding to Figure 5.

(A-C) Quantitative RT-PCR comparison of *Tubb3*, *NeuN*, *Th*, *Gfap*, *Cnp*, *Itgam* and *Cdkn1c* transcript abundance in dissected adult striatum (**A**), cortex (**B**) and cerebellum (**C**) of mice that were exposed to LPD (orange) or CD (blue) *in utero*. Expression was normalised to *β-actin* and is plotted as fold change relative to CD. Bars show geometric mean; error bars=geometric SD; n=6 for each diet group. No significant differences were detected between CD and LPD-exposed samples, as determined by unpaired t-tests ($p>0.05$).

(D) Alternative quantitative RT-PCR comparisons of *Cdkn1c* or *DARPP-32* expression levels in midbrain samples using different controls for normalisation: *β-actin* (left) or *18S*, *Tbp*, *Gapdh* (right). Both are plotted as fold change relative to CD and bars show geometric mean; error bars=geometric SD; CD n=4, LPD n=3; two-tailed unpaired t-tests (*p=0.0349).

(E) Quantitative RT-PCR analysis of *SLC6A3* and *DRD5* using animals not previously subjected to behavioural tests. Expression was normalised to *β-actin* and is plotted as fold change relative to CD. Bars show geometric mean; error bars=geometric SD; CD n=4, LPD n=3; no significant differences were detected with two-tailed unpaired t-tests ($p>0.05$).

Supplementary Videos S1 and S2

Representative videos of CD-exposed (Supplementary Video S1) and LPD-exposed (Supplementary Video S2) adult mice during habituation on Day 2 of the 20 mg/kg cocaine treatment regime.

Supplementary Videos S3 and S4

Representative videos of CD-exposed (Supplementary Video S3) and LPD-exposed (Supplementary Video S4) adult mice following administration of 20 mg/kg cocaine on Day 2 of the treatment regime.
